# Lysosomal oxidation of LDL alters lysosomal pH, induces senescence, and increases secretion of pro-inflammatory cytokines in human macrophages[S]

**DOI:** 10.1194/jlr.M088245

**Published:** 2018-11-05

**Authors:** Feroz Ahmad, David S. Leake

**Affiliations:** Institute of Cardiovascular and Metabolic Research, University of Reading, Reading, United Kingdom

**Keywords:** low density lipoprotein, lysosomes, lipid peroxidation, antioxidants, atherosclerosis

## Abstract

We have shown that aggregated LDL is internalized by macrophages and oxidized in lysosomes by redox-active iron. We have now investigated to determine whether the lysosomal oxidation of LDL impairs lysosomal function and whether a lysosomotropic antioxidant can prevent these alterations. LDL aggregated by SMase (SMase-LDL) caused increased lysosomal lipid peroxidation in human monocyte-derived macrophages or THP-1 macrophage-like cells, as shown by a fluorescent probe, Foam-LPO. The pH of the lysosomes was increased considerably by lysosomal LDL oxidation as shown by LysoSensor Yellow/Blue and LysoTracker Red. SMase-LDL induced senescence-like properties in the cells as shown by β-galactosidase staining and levels of p53 and p21. Inflammation plays a key role in atherosclerosis. SMase-LDL treatment increased the lipopolysaccharide-induced secretion of TNF-α, IL-6, and MCP-1. The lysosomotropic antioxidant, cysteamine, inhibited all of the above changes. Targeting lysosomes with antioxidants, such as cysteamine, to prevent the intralysosomal oxidation of LDL might be a novel therapy for atherosclerosis.

The presence of lipid-laden macrophage foam cells is a characteristic feature of atherosclerosis (1). The foam cells derive the majority of their lipid from uptake of modified lipoprotein, such as aggregated or oxidized LDLs (2). Under normal conditions, receptor-mediated uptake of lipoproteins traffics the particles to lysosomes, where, at an acidic pH, the lysosomal enzymes break down the protein and lipid components of LDL to products that can transverse the lysosomal membrane (3). Modified LDL is recognized and taken up by receptors on macrophages; however, due to a lack of feedback regulation for such uptake, this leads to accumulation of cholesterol in these cells (4).

Studies that examine foam cell formation by the incubation of macrophages with modified monomeric LDL (e.g., oxidized LDL) do not fully reflect the in vivo environment, as the majority of the LDL in atherosclerotic plaques is found in an aggregated state and bound to the subendothelial matrix (5, 6).

There are many mechanisms that might explain how LDL is oxidized in the arterial wall (7). Many of these are inhibited strongly by serum or interstitial fluid (8–10), but some, for instance oxidation by myeloperoxidase (11), are relatively resistant to inhibition. Furthermore, the conventional oxidized LDL hypothesis does not explain why the large clinical trials showed no protection by antioxidants, mainly α-tocopherol, against cardiovascular disease (12).

LDL might be nonoxidatively modified and aggregated by enzymes, such as SMase, lipoprotein lipase, proteases, or secretory phospholipase A_2_ enzymes, in the extracellular space of atherosclerotic lesions (13), rapidly phagocytosed by macrophages, and delivered to lysosomes where we hypothesized that it might then be oxidized (14). In support of this view, we showed that 7 days after taking up LDL aggregated by vortexing, macrophages generated ceroid in their lysosomes. Ceroid (lipofuscin) is a polymerized pro­duct of lipid oxidation found within foam cells in atherosclerotic lesions (15). The oxidation of LDL in lysosomes is catalyzed by iron. The LDL was shown to be oxidized in lysosomes (14), rather than in culture medium, because the medium consisted of Dulbecco’s modified Eagle’s medium, which does not support LDL oxidation by cells (8), and contained serum (20% v/v), which strongly inhibits LDL oxidation. Also, “pulse-chase” experiments showed that there was an increase in lipid peroxidation in the cells in the complete absence of extracellular lipoproteins (14).

SMase is found in atherosclerotic lesions and has been proposed to be one of the key enzymes causing aggregation of LDL in the extracellular space of the lesions (16). Aggregation of LDL by SMase has been reported to cause a 10-fold increase in cholesteryl ester accumulation in macrophages compared with native LDL (17). We have shown that human LDL, when aggregated with SMase, is rapidly taken up by human macrophages and oxidized inside lysosomes (18). LDL oxidation by iron at lysosomal pH is not inhibited effectively by α-tocopherol (19). Cysteamine is a drug that accumulates many fold in lysosomes, due to increased protonation of its amine group at acidic pH (20). We have shown that cysteamine is able to greatly inhibit LDL oxidation by ferrous iron under lysosomal conditions (21) and by copper ions (results not shown).

de Duve’s group (22) showed that cholesterol accumulates in lysosomes in atherosclerotic lesions. Hoff’s group (23) later showed that oxidized LDL inactivates the lysosomal protease, cathepsin B, at low pH, probably because aldehydes on oxidized LDL covalently bind to cysteine and histidine residues on cathepsin B (24, 25). This might help to explain why oxidized LDL is not degraded efficiently by lysosomes. In addition, lysosomal cholesterol and cholesteryl esters derived from oxidized LDL are resistant to removal from lysosomes (26). Oxidized cholesteryl ester aldehydes can react with lysine residues of proteins and might be involved in ceroid formation (27). Lysosomal dysfunction might play an important role in foam cell formation and plaque development (28). Cholesterol accumulation in lysosomes inhibits the vacuolar-ATPase proton pump and increases the pH of lysosomes beyond the pH range of lysosomal acid lipase (29). Oxidized LDL and cholesterol crystals are able to cause profound lysosomal dysfunction in mouse macrophages through disruptions in the pH, proteolytic capacity, and membrane integrity of these organelles (30).

Atherosclerosis is also seen to be associated with biological aging, as atherosclerotic plaques show evidence of cellular senescence characterized by reduced cell proliferation, apoptosis, elevated DNA damage, epigenetic modifications, and telomere dysfunction (31). Cellular senescence is not just associated with atherosclerosis; there is growing evidence that cellular senescence may promote atherosclerosis (32–34). It is believed that oxidative stress-induced damage to cellular components, probably due to the combination of increased reactive oxygen species (ROS) and impaired antioxidant defense, is a major contributor to the aging process (35). Although it is well-established that oxidized lipoproteins and their products are able to induce ROS-dependent senescence in cells (36–38), it is not known if the lysosomal oxidation of LDL can induce senescence in human cells.

Inflammation participates in atherosclerosis during initiation and throughout all stages of plaque development (39). Expression and secretion of inflammatory cytokines, like TNF-α, IL-1β, IL-6, and MCP-1, by the cells in the arterial intima is another characteristic feature of atherosclerosis. Many studies have shown that oxidized LDL can activate macrophages, including inflammatory reactions (40), but the possible role of lysosomal oxidation of LDL is unknown.

We report here that lysosomal oxidation of aggregated LDL affects the pH of lysosomes in human macrophages by altering the lysosomal pH and induces cellular senescence and secretion of inflammatory cytokines. These effects were reversed by the lysosomotropic antioxidant, cysteamine, which we have shown inhibits the oxidation of LDL at lysosomal pH and in lysosomes of cultured macrophages (21).

## MATERIALS AND METHODS

### Materials

Chemicals and reagents used in this study were purchased from Sigma-Aldrich, Dorset, UK or Fisher Scientific Ltd., Loughborough, UK unless otherwise stated. Solutions were prepared using ultrapure water generated from a Barnstead Nanopure system. Organic solvents were HPLC or molecular biology grade.

### LDL isolation

Blood was taken from healthy volunteers after overnight fasting using EDTA (final concentration 3 mmol/l) as the anticoagulant. LDL (1.019–1.063 g/ml) was isolated from the plasma by sequential density ultracentrifugation at 4°C, as described previously (41). LDL was stored in the dark at 4°C and used within 1 month.

### Cell culture

Human macrophages or THP-1 cells were cultured under humidified 95% air/5% CO_2_ at 37°C in Gibco RPMI-1640 containing l-glutamine (0.3 g/l), penicillin (50 IU/ml), streptomycin (50 μg/ml), amphotericin B (0.95 μg/ml), and human serum or FBS (10%, v/v), respectively, unless otherwise stated. THP-1 cells were purchased from the European Collection of Cell Cultures (Salisbury, UK). THP-1 cells were incubated in RPMI-1640 (2 ml per well) containing 10% (v/v) FCS with PMA (25 ng/ml) in 12-well tissue culture plates at 1 × 10^6^ cells per well for 72 h to differentiate into macrophages. The macrophages were then washed and rested for a further 24 h before treatment with LDL. Human monocyte-derived macrophages (HMDMs) were prepared from blood donated by healthy adults using Lymphoprep™ density gradient solution (Axis-Shield, Oslo, Norway) as previously described (42). Briefly, after separation from blood cells, monocytes were incubated in RPMI medium containing 0.05% (v/v) human serum in nonadherent 6-well tissue culture plates for 40 h, then transferred to ordinary 6-well tissue culture plates with RPMI with 10% (v/v) human serum for 10 to 14 days.

### Aggregation of LDL with SMase

Native LDL was diluted to 2 mg protein/ml with a buffer containing NaCl (150 mmol/l), MgCl_2_ (10 mmol/l), and HEPES (5 mmol/l) (pH 7.4) and incubated with SMase from *Bacillus cereus* (Sigma, catalog number S9396-25UN) at 10 mU/ml (43), until the attenuance (absorbance plus light scattering) at 680 nm increased from about 0.0017 to 0.027. The SMase-aggregated LDL (SMase-LDL) was then dialyzed against phosphate buffer [NaCl, 140 mM; Na_2_HPO_4_, 8.1 mM; NaH_2_PO_4_, 1.9 mM; and EDTA, 100 μM (pH 7.4)] pretreated with washed Chelex-100 to remove contaminating transition metals (44) and sterilized with a 0.45 μm Minisart filter before use. Aggregation was confirmed by dynamic light scattering in UV grade cuvettes with a Zetasizer Nano Series particle sizer (Malvern Instruments, Worcestershire, UK).

### Lysosomal lipid peroxidation

The process of lipid peroxidation in the lysosomes of macrophages was studied by employing a fluorescent probe called Foam-LPO, recently synthesized by Zhang et al. (45) and kindly provided by Professor Y. Xiao of Dalian University of Technology, People’s Republic of China. Foam-LPO is a BODIPY derivative containing a conjugated diene group within its fluorophore structure, which behaves as a lipid peroxidation signaling unit, and a weakly alkaline tertiary amino group, which enables the probe to be protonated and hence trapped and accumulated in the lysosomes. The conjugated diene group degrades in response to lipid peroxidation causing a fluorescent spectral shift from 586 to 512 nm, which can be measured by flow cytometry. THP-1 macrophages or HMDMs (1 × 10^6^ cells per well in 12-well tissue culture plates) were incubated with prewarmed culture medium (2 ml per well) either alone or containing native LDL (200 μg protein/ml) or SMase-LDL (200 μg protein/ml) in the presence or absence of cysteamine for 24 h at 37°C. The adherent macrophages were washed three times with prewarmed PBS and then scraped into culture medium using a plastic cell scraper, treated with Foam-LPO (2 μM) in RPMI-1640 for 15 min, and finally analyzed using a BD Biosciences C6 flow cytometer. The data were analyzed using FlowJo software by determining the mean fluorescence intensity (MFI) for each condition using untreated cells as a control. The fluorescence intensity ratio of the green channel to the red channel (ratiometry) was taken as a measure of lysosomal lipid peroxidation.

### ROS detection

We also looked at the effect of SMase-LDL and cysteamine on the overall oxidative status of the macrophages by measuring ROS using the superoxide indicator, dihydroethidium (DHE) (46). THP-1 or HMDMs (1 × 10^6^ cells per well in 12-well tissue culture plates) were incubated with prewarmed culture medium (2 ml per well) either alone or containing native LDL (200 μg protein/ml) or SMase-LDL (200 μg protein/ml) in the presence or absence of cysteamine for 24 h at 37°C. The macrophages were there scraped off the plates, washed by centrifugation (5 min, 500 *g*), and treated with DHE (10 μM) for 30 min. The cells were then washed three times with FACS buffer and analyzed using a BD Biosciences C6 flow cytometer and FlowJo software by determining the MFI for each condition using untreated cells as a control.

### Assessment of lysosomal function

The lysosomal function of cells was measured using a lysosomotropic tracking dye called LysoTracker® Red DND-99 (Life Technologies), which accumulates in lysosomes due to proton trapping (47). THP-1 macrophages or HMDMs (1 × 10^6^ cells per well in 12-well tissue culture plates) were incubated with prewarmed culture medium (2 ml per well) either alone or containing native LDL (100 μg protein/ml) or SMase-LDL (100 μg protein/ml) in the presence or absence of freshly dissolved cysteamine for 72 h at 37°C, with a change of medium every 24 h. After 72 h, the macrophages were washed three times with prewarmed PBS to remove any residual LDL or cysteamine. The adherent macrophages were scraped into culture medium using a plastic cell scraper, collected into 15 ml sterile polypropylene tubes, and centrifuged at 500 *g* for 5 min at room temperature to remove cell debris. The cells were resuspended into 200 μl RPMI-1640 medium [containing 10% (v/v) FCS], transferred into a clear 96-well round bottom microplate (Greiner CellStar®), and treated with LysoTracker Red (500 nM) in RPMI-1640 for 30 min at 37°C. Cells were washed twice with HBSS, resuspended in FACS buffer, and analyzed using a BD Biosciences C6 flow cytometer. The data analysis was done using FlowJo software by determining MFI for each histogram using untreated cells as a control.

### Measurement of lysosomal pH in macrophages

Measurement of lysosomal pH in THP-1 cells was performed using a ratiometric lysosomal pH indicator dye called LysoSensor® Yellow/Blue DND-160 (Invitrogen) (48). THP-1 macrophages or HMDMs (1 × 10^5^ cells per well in a 96-well black microplate) were incubated with either no LDL or native LDL (100 μg protein/ml) or SMase-LDL (100 μg protein/ml) every 24 h for 72 h in the presence or absence of cysteamine. After 72 h, the medium containing LDL and cysteamine was washed off with PBS and the macrophages were then incubated with 5 μM LysoSensor Yellow/Blue for 30 min at 37°C under 5% CO_2_. A separate set of THP-1 macrophages or HMDMs was used to generate the pH calibration curve by a modification of the protocol established by Diwu et al. (49). THP-1 macrophages or HMDMs (1 × 10^5^ cells per well for 72 h in a 96-well black microplate) were incubated in MES buffer (5 mM NaCl, 115 mM KCl, 1.3 mM MgSO_4_, and 25 mM MES), with the pH adjusted to a range from pH 4.0 to pH 6.0. Ten minutes prior to the LysoSensor addition, the H^+^/Na^+^ ionophore, monensin, and the H^+^/K^+^ ionophore, nigericin, were added to a final concentration of 10 μM each. This allowed lysosomal pH to equilibrate with the MES buffer and facilitated the creation of a standard curve correlating pH with the fluorescence emission spectra. Both the plates were read in a FLUOstar Optima fluorometer (BMG Labtech), with excitation at 355 nm. The ratio of emission 440/535 nm was then calculated for each sample and the pH values were determined from the standard plot generated.

### Cellular senescence

Detection of senescent cells was done by using the senescence-associated β-galactosidase staining procedure described by Dimri et al. (50) and p53/p21 expression (51, 52). THP-1 macrophages or HMDMs (4,000 per well) were plated in 12-well tissue culture plates (Corning). The adherent macrophages were washed three times with prewarmed PBS and rested for 24 h. The cells were then incubated in fresh culture medium containing either no LDL, native LDL (100 μg protein/ml), or SMase-LDL (100 μg protein/ml) every 24 h for 72 h in the presence or absence of freshly dissolved cysteamine. After 72 h, the medium was removed and cells were washed twice in PBS (2 ml) at room temperature. The cells were then either stained for β-galactosidase activity or for the expression of p53 and p21. For β-galactosidase staining, cells were fixed for 3 min with 500 μl paraformaldehyde (4% w/v) per well at room temperature. The fixative was removed and the cells were washed with PBS. Cells were then exposed to 5-bromo-4-chloro-3-indolyl-D-galactopyranoside (X-gal; pH 6) (50) staining solution (600 μl per well) and samples were incubated at 37°C without CO_2_ for 18 h. The staining solution was then removed and the plates were washed once with deionized water at room temperature and then twice with methanol. The plates were allowed to air dry after the last methanol passage and the blue-stained senescent cells were visualized using a Nikon inverted phase contrast light microscope, with images taken at 10× magnification. Quantification of the blue-stained cells was done manually (i.e., a cell was either blue or not) from five distinct fields of view from each well. For p53 and p21 expression, cells were scraped from the plates and stained with p53 monoclonal antibody (1:100, BP53-12; Thermo Fisher), FITC, and p21 monoclonal antibody (1:200, R.229.6; Thermo Fisher) followed by F(ab′)2-goat anti-rabbit IgG (H+L) secondary antibody and PE-Cyanine5.5 (L43018; Thermo Fisher), and analyzed by flow cytometry.

### Cytokine secretion

We looked at the effect of lysosomal oxidation of LDL on secretion of the pro-inflammatory cytokines, TNF-α, MCP-1, IL-1β, and IL-8, using commercially available ELISA kits. TNF-α levels were analyzed using the human TNF-α ELISA Ready-SET-Go!® reagent kit (eBioscience, Cheshire, UK), while MCP-1, IL-1β, and IL-8 were measured using ELISA MAX™ Deluxe (Biolegend). THP-1 macrophages or HMDMs were incubated in fresh culture medium alone or with native LDL or SMase-LDL (both at 50 μg protein/ml) for either 12 h or 24 h. To study the effect of cysteamine, macrophages were pretreated with different concentrations of freshly dissolved cysteamine for 24 h prior to LDL addition. After incubation with LDL, the medium was removed and the wells were washed three times with warm PBS. The washed cells were then treated with fresh culture medium containing ultrapure lipopolysaccharide (LPS) derived from *Escherichia coli* (10 ng/ml) (Sigma) for 4 h to trigger cytokine production (53). The medium from each well was collected and assayed immediately using the manufacturer’s instructions.

### Measurement of conjugated dienes

SMase-LDL (50 μg LDL protein/ml) was oxidized with freshly dissolved FeSO_4_ (5 μmol/l) at 37°C in a NaCl/sodium acetate buffer (NaCl 150, mmol/l; sodium acetate, 10 mmol/L; pH 4.5) in capped quartz cuvettes and conjugated dienes were monitored in the presence or absence of cysteamine (25 μm) using a method based on that of Esterbauer et al. (54) The change in attenuance at 234 nm was measured at 37°C against reference cuvettes containing all the components except LDL. Measurements were taken at 1 min intervals in a Lambda-2 6-cell or a Lambda Bio 40 8-cell spectrophotometer with UV Winlab software.

### Loss of LDL-tryptophan fluorescence measurement

ApoB-100 contains 37 tryptophan residues that give LDL a strong fluorescence at 331 nm (E_excitation_ 282 nm). On oxidation, the LDL-tryptophan fluorescence decreases continuously indicating that the LDL is being oxidized (55). The decrease in tryptophan fluorescence was measured on a Cary Eclipse fluorescence spectrophotometer using the time-drive method at an emission wavelength of 331 nm, with excitation set at 282 nm (55). The emission and excitation slits were set at 10 nm to obtain optimal fluorescence output. LDL (50 μg LDL protein/ml) was oxidized by freshly dissolved FeSO_4_ (5 μmol/l) at 37°C in the NaCl/sodium acetate buffer (pH 4.5) in capped quartz cuvettes with or without cysteamine, and the tryptophan fluorescence was measured every 10 min.

### Statistical analysis

Unless stated otherwise, all results are expressed as the mean ± SEM of pooled data from three to five experiments, as specified in the figure legends. Comparison of two means was done using a two-tailed unpaired Student’s *t*-test. For comparing more than two means, one-way ANOVA was used followed by Tukey’s post hoc analysis to measure the level of statistical significance between groups. The level of significance of difference is indicated in the graphs as follows: *P ≤ 0.05, **P ≤ 0.01, and ***P ≤ 0.001. ANOVA and post hoc analyses were carried out with GraphPad Prism 4 software (La Jolla, CA). A *P* value of <0.05 was taken to be a statistically significant difference.

## RESULTS

### Macrophages treated with SMase-LDL show increased lysosomal lipid peroxidation, which is inhibited by cysteamine

**Fig. 1.**
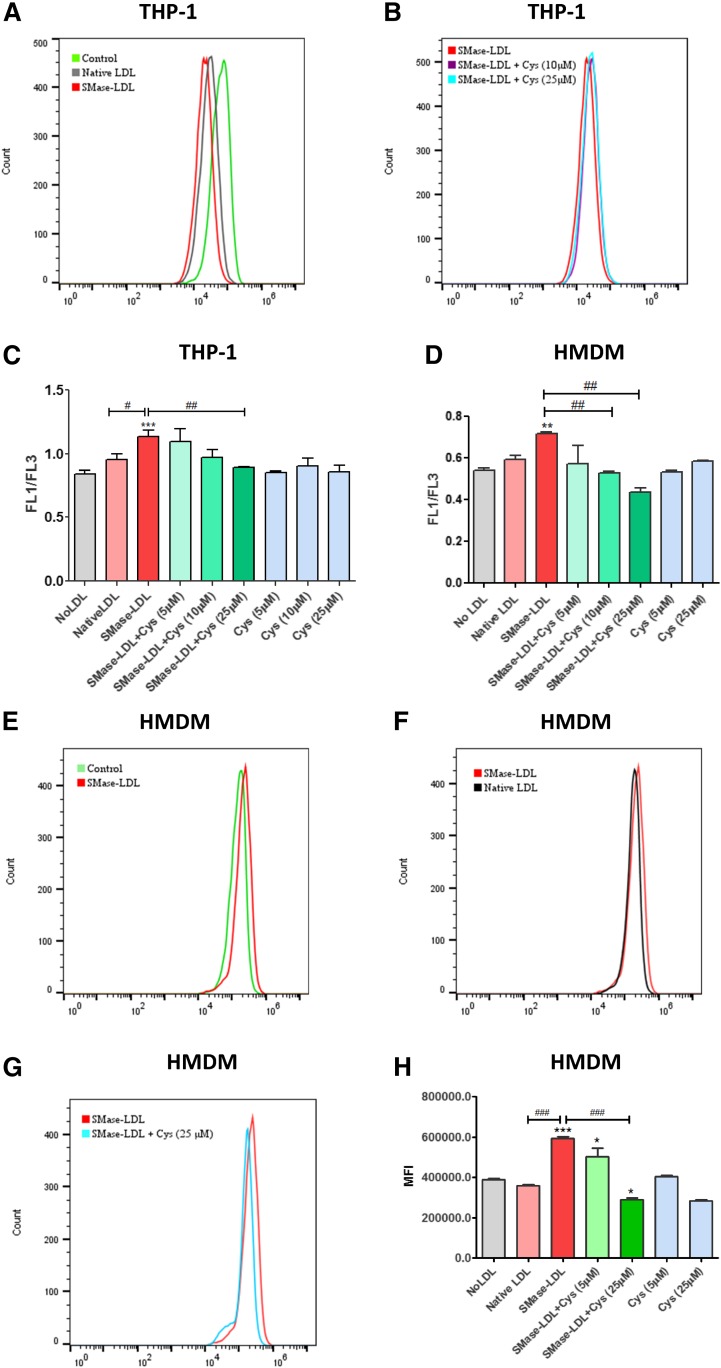
Lipid peroxidation and ROS in human macrophages. THP-1 macrophages or HMDMs were treated with no LDL, native LDL, or SMase-LDL (both at 200 μg protein/ml LDL protein) in the presence or absence of 5, 10, or 25 μM cysteamine for 24 h. The cells were then incubated with either 2 μM Foam-LPO for 15 min or 10 μM DHE for 30 min, harvested, and assayed by flow cytometry. A: MFI of of Foam-LPO in red channel of healthy THP-1 macrophages, native-LDL-treated THP-1 macrophages, and SMase-LDL-treated THP-1 macrophages. B: MFI of Foam-LPO in red channel of SMase-LDL-treated THP-1 macrophages in the presence or absence of cysteamine (Cys) (10 and 25 μM). C, D: Lipid peroxidation calculated from the ratio between the MFI of the green channel (FL1) and red channel (FL3) in THP-1 macrophages (C) and HMDMs (D). E, F: Overall ROS production in HMDM control, native LDL-treated, and SMase-LDL-treated cells. G: The effect of cysteamine (25 μM) on SMase-LDL ROS production. H: Analysis of MFI of ROS generation. ***P < 0.001, **P < 0.01 compared with untreated cells, ^###^*P* < 0.001, ^##^*P* < 0.01, ^#^*P* < 0.05; ANOVA followed by Tukey’s test; n = 3–6 independent experiments.

Non-enzymatic oxidation of LDL is considered to be a free radical-driven lipid peroxidation chain reaction (56) and, therefore, lipid peroxidation might be one of the major pathological mechanisms involved in atherosclerosis. Lysosomal lipid peroxidation was quantified in macrophages using the novel probe, Foam-LPO (45). THP-1 macrophage-like cells or HMDMs that were treated with SMase-LDL showed decreased fluorescence intensity in the red channel during flow cytometry compared with control macrophages (**Fig. 1A**), showing that lipid peroxidation was taking place in the lysosomes. Cysteamine (10 or 25 μM) significantly decreased (but not complete decrease) the red channel fluorescence with SMase-LDL (Fig. 1B). The process of lipid peroxidation was quantified by ratiometric analysis of the fluorescence intensities of the green and red channels (Fig. 1C) (45). The macrophages that were treated with SMase-LDL showed a significant increase in the lipid peroxidation levels compared with control macrophages. The lysosomotropic antioxidant, cysteamine (10 or 25 μM), reduced lipid peroxidation in the SMase-LDL-treated macrophages in a concentration-dependent manner. Cysteamine on its own had no significant effect on lipid peroxidation.

### Macrophages treated with SMase-LDL show increased ROS production, which is inhibited by cysteamine

We looked at the overall oxidative status of the HMDMs by using DHE, which detects superoxide and hydrogen peroxide. The macrophages that were treated with SMase-LDL showed increased ROS production compared with the control (Fig. 1E) and native LDL-treated macrophages (Fig. 1F). Cysteamine prevented the increase in ROS production in the macrophages that were treated with SMase-LDL (Fig. 1G) back to control levels in a concentration-dependent manner (Fig. 1H). Cysteamine on its own showed no marked effect on the total oxidative status of the macrophages (Fig. 1H).

### Lysosomal oxidation of SMase-LDL increases the lysosomal pH in macrophages

**Fig. 2.**
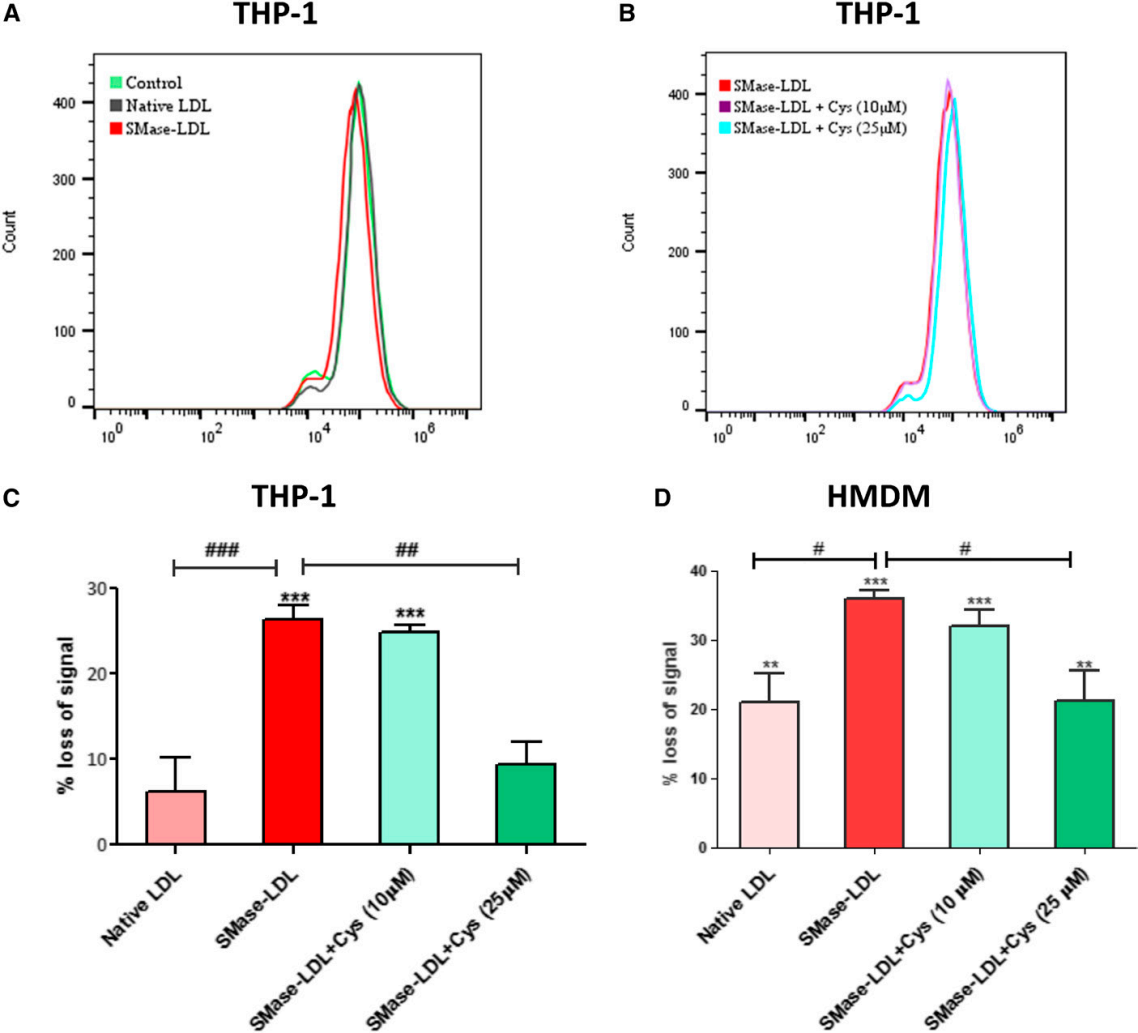
Effect of SMase-LDL and cysteamine (Cys) on LysoTracker Red accumulation by macrophages. THP-1 macrophages or HMDMs (1 × 10^6^) were cultured in 12-well tissue culture plates in RPMI medium (containing 10% v/v FCS) alone or containing native LDL or SMase-LDL with or without cysteamine (10 or 25 μM) for 72 h. All LDL concentrations were 100 μg protein/ml. After 72 h, cells were treated with 500 nM LysoTracker Red for 30 min and then assayed by flow cytometry. The MFI peak of LysoTracker Red in the red channel was then measured. A: MFI in red channel of healthy THP-1 macrophages, native-LDL-treated macrophages, and SMase-LDL-treated macrophages. B: MFI in red channel of SMase-LDL treated THP-1 macrophages in the presence or absence of cysteamine (10 and 25 μM). C: Data expressed as percentage loss of MFI of LysoTracker Red in the red channel compared with untreated control macrophages in THP-1 macrophages. D: Data expressed as percentage loss of MFI of LysoTracker Red in the red channel compared with untreated macrophages in HMDMs. **P < 0.01, ***P < 0.001 compared with untreated cells; ^#^*P* < 0.05, ^##^*P* < 0.01, ^###^*P* < 0.001 compared with SMase-LDL-treated cells; ANOVA followed by Tukey’s test; n = 4 independent experiments.

Having shown that SMase-LDL increased lysosomal lipid peroxidation, we investigated the accumulation of the lysosomotropic dye, LysoTracker Red, in macrophages. Native LDL-treated THP-1 cells showed a nonsignificant 6 ± 4% decrease in LysoTracker Red signal after 72 h compared with the control cells, whereas macrophages treated with SMase-LDL showed a significant decrease of 26 ± 2% (*P* < 0.001) in the signal compared with the control cells (**Fig. 2A**–C). In HMDMs, native LDL caused a significant 21 ± 4% (*P* < 0.01) decrease in LysoTracker Red signal compared with untreated cells, whereas in the cells treated with SMase-LDL, there was a 32 ± 2% (*P* < 0.001) loss in signal compared with the control cells (Fig. 2D). The loss in signal due to SMase-LDL was largely reversed by 25 μM cysteamine in both cell types (Fig. 2C, D). Furthermore, cysteamine on its own did not have any significant effect on LysoTracker Red accumulation (supplemental Fig. S1). We next investigated to determine whether the decreased uptake of LysoTracker Red was due to a change in the pH of the lysosomes.

**Fig. 3.**
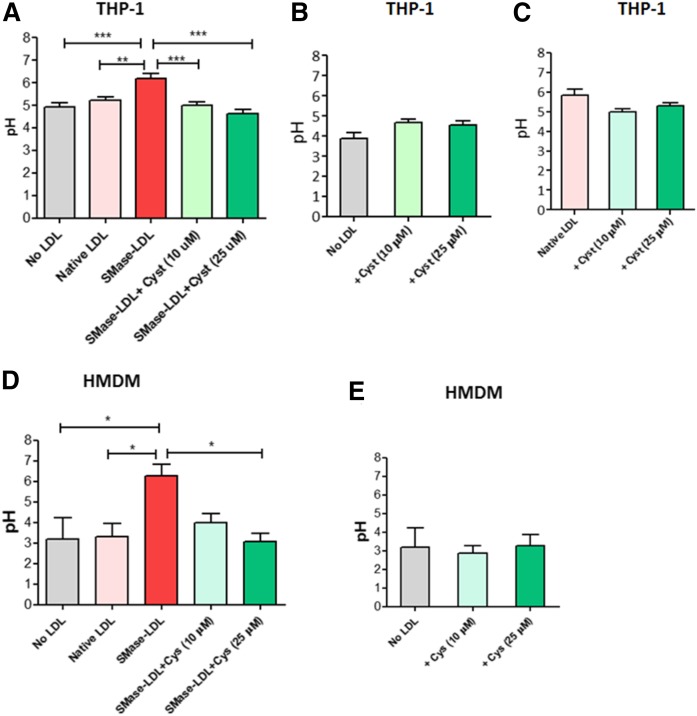
Effect of lysosomal oxidation of SMase-LDL on the pH of lysosomes in THP-1 macrophages. THP-1 macrophages (A) or HMDMs (D) were cultured in a black 96-well microplate at 1 × 10^5^ per well in RPMI medium (containing 10% v/v FCS) with no LDL, native LDL, or SMase-LDL (both at 100 μg protein/ml) with or without cysteamine (Cyst/Cys) (10 or 25 μM) for 72 h. The cells were then treated with 5 μM LysoSensor Yellow/Blue for 30 min at 37°C. The samples were then read in a FLUOstar Optima fluorometer, with excitation at 355 nm. The ratio of emission at 440 nm and 535 nm was then calculated for each sample and the pH values determined from a standard plot. B, E: The effect of cysteamine on control THP-1 macrophages (B) and HMDMs (E). C: The effect of cysteamine on native LDL-treated THP-1 cells. *P < 0.05, **P < 0.01, and ***P < 0.001 compared with SMase-LDL-treated macrophages; ANOVA followed by Tukey’s test of at least four independent experiments.

Native LDL did not significantly increase the acidic pH of the lysosomes (**Fig. 3A**, D). Treatment of THP-1 cells and HMDMs with SMase-LDL for 72 h significantly increased the lysosomal pH to 6.2 ± 0.2 in THP-1 macrophages and to 6.3 ± 0.6 in HMDMs. Cysteamine treatment (10 or 25 μM) prevented the SMase-LDL-induced increase in lysosomal pH in both types of cells (Fig. 3A, D). To determine whether the effect of cysteamine on lysosomal pH was due to inhibition of lysosomal oxidation of LDL or due to a direct effect on lysosomal pH, the effect of cysteamine was assessed in untreated or native LDL-treated THP-1 macrophages. Treatment with 10 or 25 μM of cysteamine had no significant effect on the lysosomal pH in either case (Fig. 3B, C). Also, cysteamine (10 or 25 μM) did not have any significant effect on HMDMs on its own (Fig. 3E).

### Lysosomal oxidation of SMase-LDL induces senescence in macrophages

**Fig. 4.**
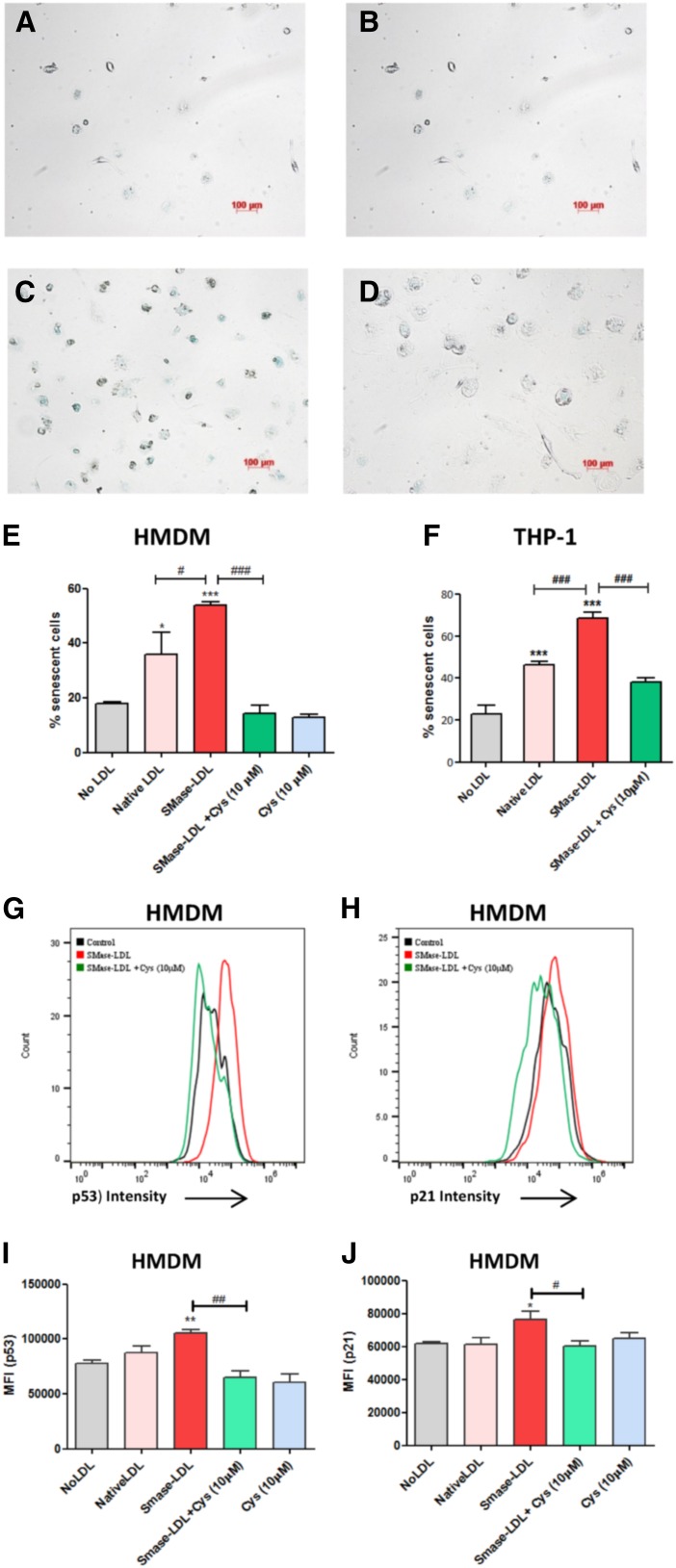
Effect of lysosomal oxidation of LDL on senescence in human macrophages. HMDMs were cultured in 12-well tissue culture plates at 3,000 cells per well in RPMI medium (containing 10% v/v lipoprotein-deficient serum) containing either no LDL (A), native LDL (B), SMase-LDL alone (C), or SMase-LDL (D) (all at 100 μg protein/ml) with 10 μM cysteamine (Cys) for 72 h. The cells were then stained to identify any senescent cells by a lysosomal β-galactosidase activity assay and p53 and p21 expression. E, F: The percentage of senescent cells in HMDMs (E) and THP-1 cells (F), which had been treated in the same way. The images shown are representative of three independent experiments. G, H: The MFI for p53 (G) and p21 (H) expression in HMDMs. I, J: A comparison of p53 (I) and p21 (J) MFI under various treatment conditions. *P < 0.05, **P < 0.01, and ***P < 0.001 compared with the control cells; ^#^*P* < 0.05, ^##^*P* < 0.01, and ^###^*P* < 0.001 for the indicated comparison; ANOVA followed by Tukey’s test of at least three independent experiments.

As cell senescence might be important in atherosclerosis (33), we investigated the effect of lysosomal LDL oxidation on the lysosomal senescence-associated β-galactosidase assay and the expression p21 and p53 proteins. Incubation of THP-1 cells or HMDMs with native LDL and, especially, SMase-LDL increased the senescence-associated β-galactosidase activity in their lysosomes (**Fig. 4A**–F, supplemental Fig. S2). Cysteamine (10 μM) treatment reduced β-galactosidase activity substantially. We then looked at the expression of two other senescent markers, p53 and p21, in HMDMs and found that treatment with SMase-LDL significantly increased these markers compared with untreated control cells, while treatment with cysteamine significantly reduced the SMase-LDL-induced expression of both of these markers (Fig. 4G–J). Cysteamine on its own had no effect on p53 or p21 expression.

### Lysosomal oxidation of SMase-LDL leads to increased secretion of pro-inflammatory cytokines

**Fig. 5.**
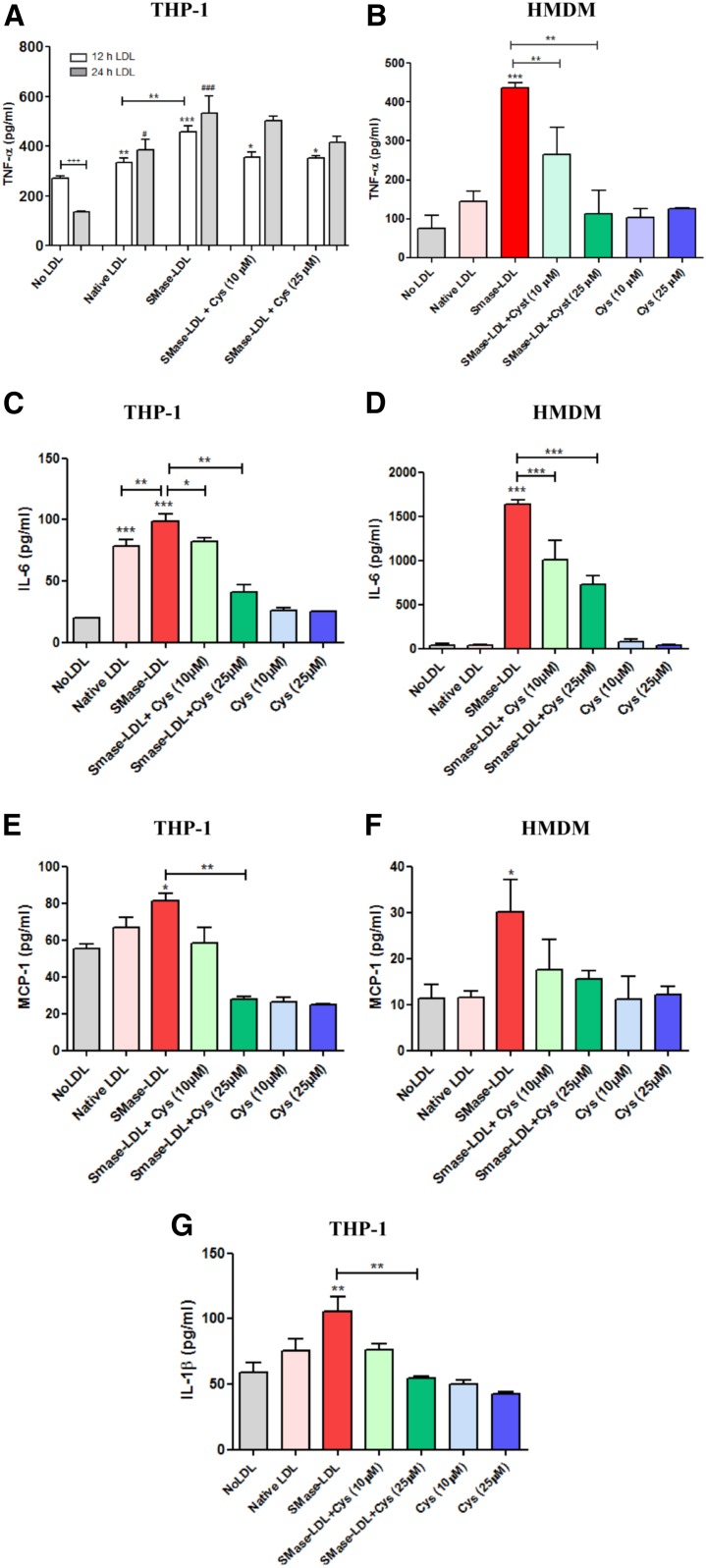
Effect of SMase-LDL on cytokine expression in macrophages. THP-1 macrophages or HMDMs were incubated in fresh RPMI-1640 medium (containing 10% v/v FBS), alone or with native LDL or SMase-LDL (both at 50 μg protein/ml) for either 12 or 24 h, and the medium was assayed for various pro-inflammatory cytokines. Some of the wells were preincubated with cysteamine (Cys) (10 or 25 μM) for 24 h prior to SMase-LDL treatment. After SMase-LDL treatment, the cells were washed with PBS and then stimulated with LPS (10 ng/ml) for 4 h at 37°C, and the medium was collected and assayed for cytokine levels. *P < 0.05, **P < 0. 01, and ***P < 0.001 compared with the control cells; ^#^*P* < 0.05, ^##^*P* < 0.01, and ^###^*P* < 0.001 for the indicated comparison. The data shown are from at least three independent experiments and were analyzed by one-way ANOVA followed by Tukey’s posttest.

Inflammation, in addition to cell senescence, is important in atherosclerosis (39). We therefore investigated to determine whether the lysosomal oxidation of LDL might cause an increase in secretion of inflammatory cytokines. THP-1 macrophages showed a significant increase in LPS-stimulated TNF-α secretion after both 12 and 24 h incubation with native LDL and more so with SMase-LDL (**Fig. 5A**). Secretion of TNF-α tended to increase, but not significantly, from 12 h to 24 h with native LDL and SMase-LDL treatment, but decreased in the control cells, which received no LPS. In HMDMs, there was a large increase in TNF-α secretion when treated with SMase-LDL for 24 h, whereas native LDL had no significant effect (Fig. 5B). Prior treatment with cysteamine for 24 h reduced the secretion of TNF-α by the macrophages incubated with SMase-LDL in a concentration-dependent way, suggesting that the increased secretion seen with SMase-LDL was due to the lysosomal oxidation of LDL. Furthermore, cysteamine on its own did not have any effect on LPS-induced TNF-α secretion in HMDMs. Similar effects of SMase-LDL and cysteamine were seen for IL-6, IL-1β, and MCP-1 secretion (Fig. 5C–G).

### Cysteamine inhibits LDL oxidation by iron at lysosomal pH

**Fig. 6.**
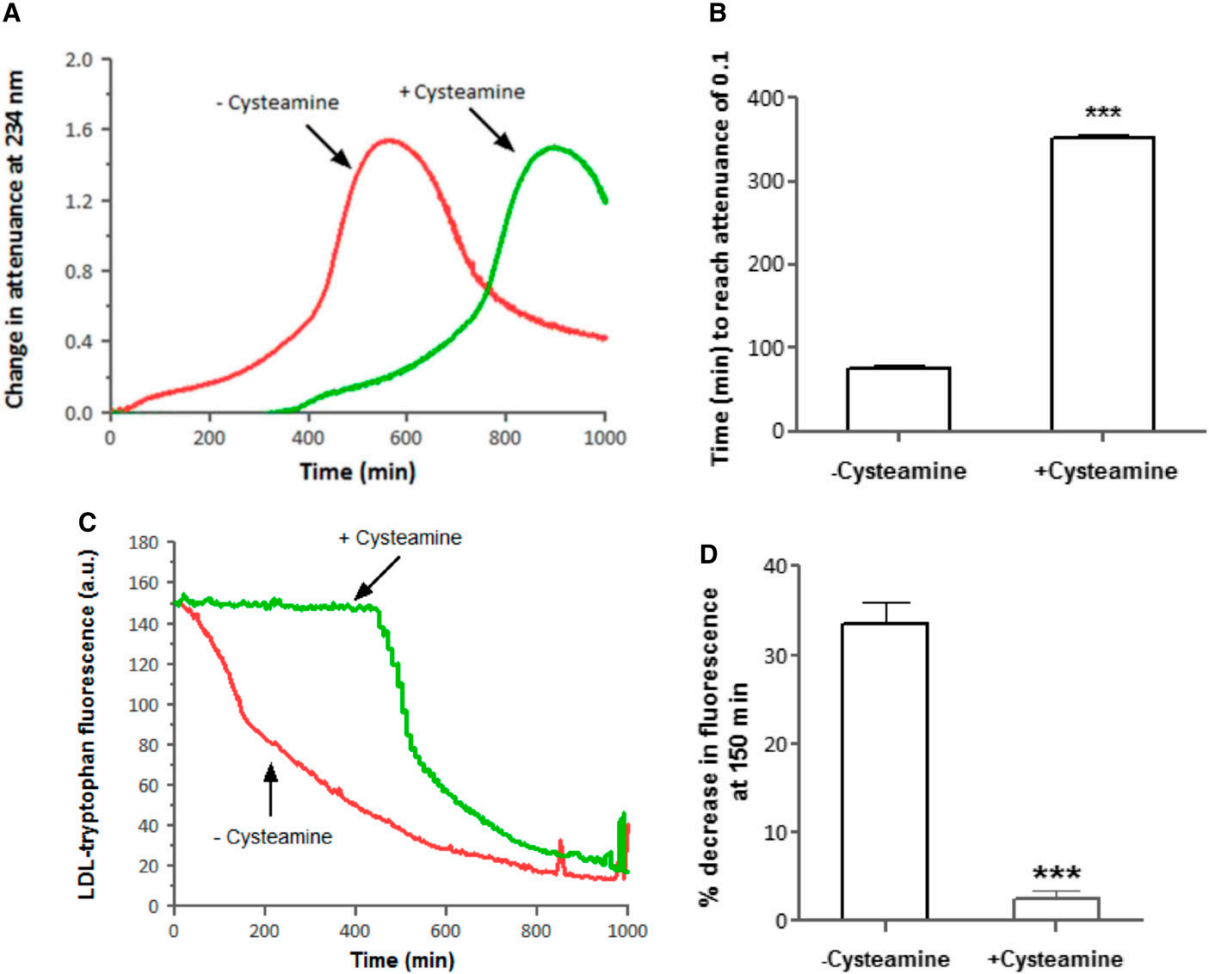
Effect of cysteamine on LDL oxidation catalyzed by iron at pH 4.5. SMase-LDL (50 μg protein/ml) in NaCl/sodium acetate buffer (pH 4.5) was incubated with 5 μM FeSO_4_ in the presence or absence of cysteamine (25 μM) at 37°C in capped quartz cuvettes. Oxidation was monitored by measuring the change in attenuance at 234 nm (A) or loss of LDL-tryptophan fluorescence against appropriate reference cuvettes (C). This is a representative example of three independent experiments. B: Time taken to reach an attenuance of 0.1 during the oxidation with iron. D: Decrease in LDL-tryptophan fluorescence after 150 min of oxidation. ***P < 0.001; *t*-test; n = 3 independent experiments.

We have previously shown that catalytically active iron within the lysosomes causes LDL oxidation. Cysteamine (25 μM) completely inhibited the initial oxidation of SMase-LDL by ferrous iron in vitro in a spectrophotometer and caused a significant increase in the lag phase at pH 4.5 (**Fig. 6A**). The time taken for SMase-LDL to reach an attenuance of 0.1 during oxidation catalyzed by iron was 76 ± 3 min when no cysteamine was added, compared with 352 ± 5 min in the presence of cysteamine (*P* < 0.001, n = 3), which is a 5 ± 0.2-fold inhibition of LDL oxidation (Fig. 6B). Incubation of SMase-LDL with ferrous sulfate led to continuous loss of tryptophan fluorescence, with a sharp loss initially (Fig. 6C). Cysteamine (25 μM) significantly prevented the loss of LDL-tryptophan fluorescence for 500 ± 50 min. The LDL fluorescence decreased by 34 ± 3% after 150 min of LDL oxidation with ferrous iron, whereas in the presence of cysteamine, the fluorescence decreased by only 2 ± 0.9% (*P* < 0.001, n = 5) (Fig. 6D).

## DISCUSSION

The lysosomal cholesteryl esterase and proteases that degrade LDL are normally active at about pH 4.5 (57, 58). A change in lysosomal pH can cause lysosomal dysfunction (59). Lysosomes generate and maintain their pH gradients by using the activity of a proton-pumping V-type ATPase, which uses the metabolic energy of ATP to pump protons into the lysosomal lumen (60). The lysosome receives extracellular cargo (via endocytosis) and cytoplasmic material (via autophagy) for degradation (61). Failure of the lysosome to process its contents efficiently leads to an accumulation of undigested material inside the lumen and can cause lysosomal dysfunction (62).

The accumulation of lysosomal free cholesterol has been shown to directly cause an increase in lysosomal membrane cholesterol content (29). The data presented here have shown that treatment of human macrophages with SMase-LDL for 3 days decreased LysoTracker Red accumulation (Fig. 2). The lysosomotropic antioxidant, cysteamine, attenuated this loss. The decrease in LysoTracker Red accumulation was probably due to an increase in lysosomal pH. SMase-LDL treatment for 72 h increased the lysosomal pH of the THP-1 macrophages considerably from 4.9 to 6.2 (which represents a decrease in hydrogen ion concentration of 20 times), compared with the pH of untreated cells, and from 3.2 to 6.3 in HMDMs, a decrease in hydrogen ion concentration of 1,250 times (Fig. 3D). The increase in pH would be expected to substantially decrease the degradation of endocytosed LDL (57, 58) and lead to more lipid accumulation in lysosomes and thus more lipid-laden foam cells. Cysteamine prevented the SMase-LDL-induced increase in lysosomal pH, but had no great effect on the lysosomal pH of macrophages in the absence of LDL or in the presence of native LDL. Importantly, this suggests that the increase in lysosomal pH in the presence of SMase-LDL was due to the lysosomal oxidation of this LDL, possibly due to lipid peroxidation products, such as 7-ketocholesterol, 4-hydroxynonenal, or malondialdehyde, which have previously been shown to inhibit the activity of the lysosomal V-ATPase causing lysosomal dysfunction (29, 63, 64).

We found that both THP-1 macrophages and HMDMs that were treated with SMase-LDL had increased lipid peroxidation levels in their lysosomes, and cotreatment with cysteamine reduced the lysosomal lipid peroxidation in a concentration-dependent manner (Fig. 1A–D). Furthermore, we found that SMase-LDL-treated HMDMs had higher ROS levels compared with control and native LDL-treated macrophages, and cysteamine considerably reduced the ROS levels. We have recently proposed that hydroperoxyl radicals (protonated superoxide radicals, HO_2_^•^) are the main species in the lysosomes of macrophages that can oxidize LDL (21) and these might possibly be responsible for the increased ROS in the presence of SMase-LDL.

There is strong evidence suggesting that decreased lysosomal proteolytic activity and increased lysosomal pH occur as a consequence of aging in long-lived post mitotic cells (65–67). In fact, increasing lysosomal function is being considered as a plausible avenue for anti-aging interventions so as to increase the longevity of cells (68). It has been proposed that oxidative stress-induced damage to cellular components, probably due to the combination of higher levels of ROS and impaired antioxidant defense, is the main contributor to the aging process (35). The accumulation of oxidation products of cholesterol (oxysterols) has been seen to induce senescence in human cells through the generation of ROS (68, 69). Native LDL and, especially, SMase-LDL treatment for 3 days induced senescence in human macrophages (Fig. 4), and treatment with cysteamine significantly decreased the senescence induced by SMase-LDL, suggesting that the senescence was due to lysosomal oxidation of SMase-LDL (and native LDL). The significance of this is uncertain, however, as macrophages in vivo (unlike the tumor cell line THP-1 macrophages) are not considered to be long-lived cells.

Inflammation plays a key role in the initiation, progression, and rupture of atherosclerotic lesions (39). Both minimally oxidized LDL and more highly oxidized LDL cause the secretion of pro-inflammatory cytokines by macrophages (70, 71) by activating toll-like receptor-4 (TLR-4). Some studies have shown, however, that oxidized LDL inhibits the production of inflammatory cytokines by macrophages in response to inflammatory stimuli, such as LPS (72). The effect of vortexed and acetylated LDL on the expression of the pro-inflammatory cytokine, TNF-α, is controversial, with some reports showing a decrease in its levels (73, 74) and others showing an increase (77). LPS is considered to be a classical ligand of TLR-4 receptors (76). We sought to determine whether lysosomal oxidation of SMase-LDL by human macrophages could alter the secretion of pro-inflammatory cytokines (TNF-α, IL-1β, IL-6, and MCP-1). Both native LDL and, to a greater extent, SMase-LDL increased the LPS-induced secretion of all the cytokines (Fig. 5). The potentiation of LPS-induced TNF-α secretion by native LDL is in agreement with the previous studies by Netea et al. (77). Preincubation with cysteamine decreased the secretion of these pro-inflammatory cytokines by macrophages incubated with SMase-LDL, suggesting that some of the secretion was due to the lysosomal oxidation of LDL. The reduction by cysteamine was sometimes only partial, probably because LPS was directly stimulating cytokine secretion. The increased secretion of pro-inflammatory cytokines by native and SMase-LDL might possibly be due to the oxidative stress caused by the lysosomal oxidation of LDL (78), but the exact mechanism needs detailed investigation.

In conclusion, we have shown that the lysosomal oxidation of LDL alters the function of macrophages in potentially atherogenic ways, namely, an increase in lysosomal pH, cell senescence, and pro-inflammatory cytokine secretion. These effects can be inhibited by the lysosomotropic antioxidant, cysteamine, suggesting a novel therapeutic approach to treat atherosclerosis.

## Supplementary Material

Supplemental Data
