# Supplementary material for: Lysosomal oxidation of LDL alters lysosomal pH, induces senescence, and increases secretion of pro-inflammatory cytokines in human macrophages
Source: J Lipid Res. 2018 Nov 5;60(1):98–110. doi: 10.1194/jlr.M088245 (PMC6314264; doi:10.1194/jlr.M088245)
Supplement: Supplemental Data [file supp_60_1_98__index.html]

Lysosomal oxidation of LDL alters lysosomal pH, induces senescence and increases secretion of pro-inflammatory cytokines in human macrophages — Lysosomal oxidation of LDL alters lysosomal pH, induces senescence, and increases secretion of pro-inflammatory cytokines in human macrophages — Supplemental Data 

# Lysosomal oxidation of LDL alters lysosomal pH, induces senescence, and increases secretion of pro-inflammatory cytokines in human macrophages

## Supplemental Data

- Supplemental Figure S1 (.pdf, 15 KB) - Supplemental Figure S1
- Supplemental Figure S2 (.pdf, 306 KB) - Supplemental Figure S2
