# Supplementary material for: Lysosomal oxidation of LDL alters lysosomal pH, induces senescence, and increases secretion of pro-inflammatory cytokines in human macrophages
Source: J Lipid Res. 2018 Nov 5;60(1):98–110. doi: 10.1194/jlr.M088245 (PMC6314264; doi:10.1194/jlr.M088245)
Supplement: Supplemental Data [file 10.1194_M088245_jlr.M088245-1.pdf]

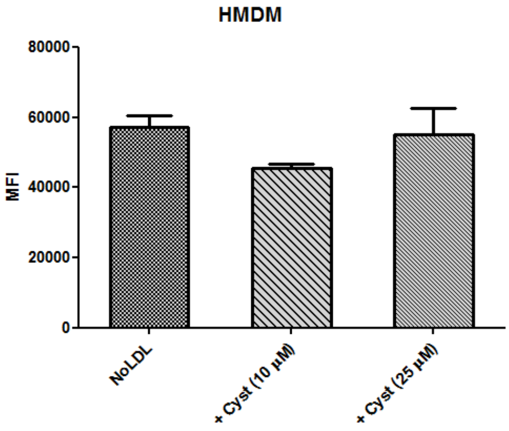

Supplemental Figure S1 Effect of cysteamine on lysosomal function

HMDM (1 x 10^6) were cultured in 12 well tissue culture plates in RPMI medium (containing 10% v/v FCS) with cysteamine (10 µM or 25 µM) for 72 h. The cells were then washed and treated with 500 nM Lysotracker Red for 30 min and then assayed by flow cytometry. Mean fluorescence intensity peak of Lysotracker Red in the red channel was then measured. Mean of 3 independent experiments.
