# Supplementary material for: Lysosomal oxidation of LDL alters lysosomal pH, induces senescence, and increases secretion of pro-inflammatory cytokines in human macrophages
Source: J Lipid Res. 2018 Nov 5;60(1):98–110. doi: 10.1194/jlr.M088245 (PMC6314264; doi:10.1194/jlr.M088245)
Supplement: Supplemental Data [file 10.1194_M088245_jlr.M088245-2.pdf]

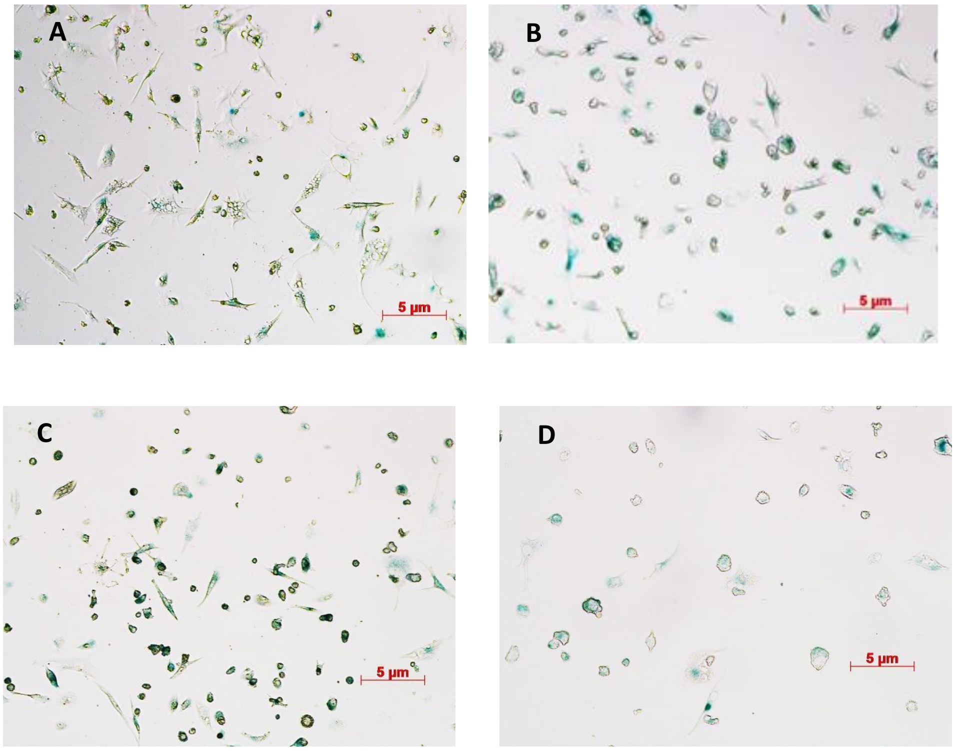

Supplemental Figure S2 Effect of lysosomal oxidation of LDL on senescence in THP-1 macrophages

THP-1 cells were cultured in 12 well tissue culture plates at 3000 cells per well in RPMI medium (containing 10% v/v lipoprotein-deficient serum) containing either no LDL (A), native LDL (B), SMase-LDL alone (C) or SMase-LDL (all at 100 µg protein/ml) with 10 µM cysteamine (D) for 72 h. The cells were then stained to identify any senescent cells by a lysosomal β-galactosidase activity assay. The images shown are representative of three independent experiments.
